# Supplementary material for: The female side of pharmacotherapy for ADHD—A systematic literature review
Source: PLoS One. 2020 Sep 18;15(9):e0239257. doi: 10.1371/journal.pone.0239257 (PMC7500607; doi:10.1371/journal.pone.0239257)
Supplement: S1 Table — (DOCX) [file pone.0239257.s001.docx]

**S1 Table. Sex differences in ADHD pharmacotherapy prescription rates.**

| **Author (year) / study aim** | **Sample characteristics (composition, sex)** | **Medication prescribed** | **Age range in years (M)** | **Timeframe** | **Study design** | **Sex-related results on prescription rates** |
| --- | --- | --- | --- | --- | --- | --- |
| **[64] Barbaresi et al. (2006)**  Study aim: To examine rates of stimulant treatment, treatment effectiveness, and occurrence of side effects, as well as report on variations in treatment rates and effects by gender and presentation. | ADHD  F 95  M 284 | MPH  DexAMP  LevoAMP-DexAMP, Pemoline  MethAMP | Up to 17.2 (10.4)  (Longitudinal) | 1976 – 1982 birth cohort | Observational population-based (retrospective registry study) | **MPH**  F < M (55.8% vs 69.7%), *d* = 0.33  **All other medication**  F = M  **No medication**  F > M (28.7% vs 18.7%), *d* = 0.3 |
| **[65] Klein et al. (2016)**  Study aim: Compare pattern of prescriptions in adolescents with ADHD for Oregon Medicaid patients. | ADHD  F 2.876  M 7.877 | MPH,  AMP-dexAMP mixed salts, LisdexAMP  dexMPH,  dexAMP  Guanfacine  Clonidine, ATX | 10.8 (3.4) (age of first prescription) | 2012-2013 | Population-based  (limited data set review of Medicaid claims | **All medication**  F < M (25.2% vs 74.8%) |
| **[66] McCabe et al. (2016)**  Study aim: To examine the effect of age of initiation, length and medication kind on ADHD and substance abuse. | General population  F 20.986  M 19.372  3.539 stimulant users  1.332 non-stimulant users | MPH  dexAMP  AMP  ATX  Guanfacine  Bupropion  Modafinil | Modal age: 18 | 2005-2014 | Population-based  (surveys of cross-sectional, nationally representative sample of high school seniors in the US) | **Stimulants**  F < M (2.1% vs 4.2%)  **Non-stimulants**  F = M (3.1% vs 3.5%) |
| **[67] Song & Shin (2016)**  Study aim: To examine prescribing patterns for ADHD medications among children and adolescents in Korea. | 2007  F 14699  M 58005  2009  F 14.803  M 59.160  2011  F 17.981  M 67.487 | MPH  ATX | 1 – 17 (10.14-11.19) | 2007 to 2011 | Population-based  (retrospective medical insurance database study) | **MPH**  2007  F < M: 70.31% vs 74.8%  2011  F < M: 67.47% vs 71.1%  **ATX**  2009  F < M: 4.5% vs 6.1%  2011  F < M: 10.67% vs 13.7% |
| **[68] Chang et. al. (2016)**  Study aim: To explore the relationship between ADHD medication and depression. | ADHD  F 12.503  M 26.249 | MPH  AMP  dexAMP)  ATX | 8 – 46 (NR) in 2006 | Medication use in 2006-2008  Occurrence of depression in 2009 | Population-based (retrospective registry study) | **All medication**  8-15 years: F < M (34.9% vs 56.1%)  16-25 years: F > M (32.1% vs 25.6%) 26-35 years: F > M (16.2% vs 10.2%) 36-46 years: F > M (16.8% vs 9.1%) |
| **[69] Zoega et al. (2011)**  Study aim: To explore the  accessibility of ADHD drugs and the prevalence of their use among children, adolescents and adults in the five Nordic countries; Denmark, Finland, Iceland, Norway and Sweden. | General population  24.919.145  ADHD medication users  Total 68 776  MPH 57.273  ATX 8.280  Other 3223 | MPH  AMP  dexAMP  Modafinil  ATX | Age range not specified but categorisations from birth to age 27+ years | 2007 | Population-based (retrospective prescription database study) | **All medication**  Medication use prevalence ratio F:M  Age group 7-15 1:2.18  All ages 1:4.48  *Percentages could not be calculated* |
| **[70] Karlstad et al. (2016)**  Study aim: To investigate use of ADHD drugs (stimulants and atomoxetine) among the entire adult population in the Nordic European countries. | General population  F 34.446  M 42.450 | MPH  ATX  AMP  dexAMP | 18 – 64 (NR) | 2008–2012 | Population-based (retrospective prescription register study) | **All medication**  18-24 years: F < M (0.93% vs 1.19%)  25-34 years: F < M (0.60% vs 0.73%)  35-44 years: F < M (0.48% vs 0.51%)  45-64 years: F < M (0.18% vs 0.21%) |

ADHD: Attention Deficit Hyperactivity Disorder, ADHD-C: Attention Deficit Hyperactivity Disorder – Combined presentation, ADHD-HI: Attention Deficit Hyperactivity Disorder – Hyperactive-Impulsive presentation, ADHD-I: Attention Deficit Hyperactivity Disorder – Inattentive presentation, AMP: Amphetamine, ATX: Atomoxetine, *d* = Cohen’s *d*, dexAMP: Dextroamphetamine, dexMPH: Dextroamphetamine, F: Females, M: Males, MethAMP: Methylamphetamine, MPH: Methylpheniate, LevoAMP: LevoAmphetamine; LisdexAMP: Lisdextroamphetamine
