# Supplementary material for: The female side of pharmacotherapy for ADHD—A systematic literature review
Source: PLoS One. 2020 Sep 18;15(9):e0239257. doi: 10.1371/journal.pone.0239257 (PMC7500607; doi:10.1371/journal.pone.0239257)
Supplement: S2 Table — (DOCX) [file pone.0239257.s002.docx]

**S2 Table. Summary of included studies on effects of pharmacotherapy on females and males with ADHD.**

| **Author (year) / study aim** | **Sample characteristics(sex; symptom presentation)** | **Comparison sample characteristics (sex; symptom presentation)/** | **Age range in years (M)** | **Treatment chracteristics (type; duration)** | **Study design** | **Adverse events** | **Sex-related results on medication effects** |
| --- | --- | --- | --- | --- | --- | --- | --- |
| **[5] Robison et al., 2008**  Study aim: To compare baseline attributes of males and females with ADHD and compare their treatment responsiveness to ADHD medication on symptom severity and quality of life | ADHD  F 188  M 348  Presentation  371 ADHD-C  165 ADHD-I  12 ADHD-HI | ADHD  Same sample | NR (41.2) | ATX (10 weeks) vs  Placebo (10 weeks) | Double-blind, placebo-controlled, multi-center studies | NR | **Improvement in symptom severity, quality of life and emotional dysregulation**  Females vs males  Emotional Dysregulation: F > M, *d* = 0.28  Social life: F > M, *d* = 0.19  Improvement symptom severity:  F > M, *d* = 0.31 |
| **[64] Barbaresi et al. (2006)**  Study aim: To examine rates of stimulant treatment, treatment effectiveness, and occurrence of side effects, as well as report on variations in treatment rates and effects by sex and presentation | ADHD  F 95  M 284  Presentation:  228 ADHD-C  25 ADHD-HI  83 ADHD-I  2 not specified | Non-ADHD  965 (mixed sex, presentation n/a) | 10.4 – 17.2 | MPH, DexAMP, LevoAMP-DexAMP, Pemoline, MethAMP (various durations of treatment) | Population-based (retrospective registry study) | NR  Gender not significantly associated with  developing side effects (F: 17.5% vs M: 23.6%) | **Response to stimulants**  Females vs males  Positive response to MPH  F = M (73% vs 75%)  No response to MPH  F = M (17% vs 13%)  Positive response to dexAMP  F < M (51% vs 78.8%)  *Effect sizes could not be calculated* |
| **[68] Chang et. al (2016)**  Study aim: To explore the relationship between ADHD medication and depression. | ADHD  F 12503  M 26249  Presentation NR | ADHD  Same sample during period of no medication use | 8 – 46 (NR) | Stimulants (MPH, AMP, dexAMP), Non-stimulant (ATX)  3 months continuous medication use vs no medication use | Population-based (retrospective registry study) | NR | **Prevalence of depression**  Females vs males  F: Medicated < not medicated: *d* = 0.21  M: Medicated < not medicated: *d* = 0.22 |
| **[71] Pelham et al. (1989)**  Study aim: To investigate whether stimulant medication for ADHD has equivalent effects on boys and girls. | ADHD  F 12  Presentation:  12 ADHD-I | ADHD  M 12 | 5.5 - 11.4 (9.1) | MPH (5 weeks) vs placebo (5 weeks) | Observational study | NR | **Improvement in behaviour, academic performance and peer interaction**  Females vs males  Conduct problems  F < M, *d* = 0.281  Academic performance:  Nonsense spelling:  F < M, *d* = 1.919  Times math numbers attempted:  F < M. *d* = 1.114  Nonsense spelling  F < M, *d* = 1.919 |
| **[72] Günther, Herpertz-Dahlmann & Konrad (2010)**  Study aim: To examine sex differences in the dose-dependent influence  of MPH on attention and to investigate whether boys and girls with ADHD differ in their attentional functions. | ADHD  F 27  M 27  Presentation NR | ADHD  F 27  M 27 | 8-12 (11.05) | MPH (6 days) vs Placebo (6 days) | Double-blind,  placebo-controlled trial | NR | **Improvement in attentional functions**  Females vs males  Focused attention:  F > M: *d* = 0.96  Sustained attention:  F < M: *d* = 0.58  Omission errors:  F > M: *d* = 0.72  Divided attention:  F > M: *d* = 76 |
| **[73] Wang, Chen & Huang, 2015**  Study aim: To investigate sex differences in behavioral symptoms and neuropsychological performance in patients with ADHD treated with MPH  during 24 months in a clinical setting. | ADHD  F 26  Presentations NR | ADHD  M 128 | NR (M F: 12.8, M: 13.2) | MPH (24 months) | Nonrandomized, prospective clinical trial | NR | **Improvement in attention**  Females vs males  Improvement attention test:  F < M  - Composite score (omission errors, commission errors, response sensitivity):  F < M  - Composite score (response time, response time variability, ADHD score):  F = M  Sluggish Response improvement:  F = M  **Improvement in symptom severity**  Symptom severity improvement  F = M  Symptom severity at 24 months (parent-clinician rating)  F < M  *Effect sizes could not be calculated* |
| **[74] Karci et al. (2018)**  Study Aim: Investigate adolescents’ changes in symptoms of anxiety and depression associated with ADHD and quality of life after methylphenidate treatment. | ADHD  F 18  M 32  Presentation  39 ADHD-C  11 ADHD-I | ADHD  Same sample pre-treatment | 13-18 (14.5) | MPH (3+ months) vs pre-treatment | Retrospective patient chart screening | NR | **Self-rated improvement in quality of life**  Females  Physical functioning  MPH vs no medication: *d* =-0.54  School functioning  MPH vs no medication: *d* = 0.60  Males  Psychosocial functioning  MPH vs no medication: *d* = 0.40  School functioning  MPH vs no medication: *d* = 0.58 |
| **[75] Sheridan, Hinshaw & D’Esposito (2010)**  Study aim: To test, using fMRI and functional connectivity analyses, whether there is less PFC activity during a working memory tasks in female adolescents when on / off ADHD medication, whether accuracy is the same or better | ADHD  F 5  Presentation NR | ADHD  Same sample | 12 – 17 (14.8) | MPH, AMP vs medication-free | fMRI and functional connectivity analyses | NR | **Improvement in accuracy**  MPH or AMP > not medicated:  *d* = 0.42 |
| **[76] Sonuga-Barke et al. (2007)**  Study aim: To investigate sex differences in pharmacodynamics of MPH. | ADHD  F 48  M 136  Presentation:  151 ADHD-C  24 ADHD-I  9 ADHD-HI | ADHD  Same sample | NR (9.58) | MPH (7 days) vs placebo (7 days) | Double blind, cross-over trial | NR | **Improvement in efficacy of medication**  Females vs Males  CON  F > M: 1.5 hours (*d* = -1.26) to 12 hours (*d* = -1.05)  MCD-EQXL  F > M: 1.5 hours (*d* = -2.96) to 12 hours (*d* = -1.19)  MCD-EQXL vs CON  F (*d* = -1.09) < M (*d* =-2.32): 1.5 hours  F (*d* = -1.19) < M (*d* =-2.39): 3 hours  F (*d* = 1.10) < M (*d* = -1. 66): 3.5 hours  After 12 hours: CON superior to MCD-EQXL in both sexes, but effects smaller in females:  F (*d* = -0.62) < M (*d* = -1.24). |
| **[77] Quinn et al. (2017)**  Study aim: To investigate concurrent and long-term associations between ADHD medication treatment and substance-related events. | ADHD  F 1,414,183  Presentation NR | ADHD  M 1,579,704 | F: 19 – 42 (28)  M: 15 – 34 (21) | Stimulants: AMP, dexAMP, dexMPH, lisdexAMP, MPH  Non-stimulant: ATX (1 – 120 months) | Retrospective registry study of US healthcare claims | NR | **Risk of SREs related to stimulant and non-stimulants use**  Females vs males  Odds of concurrent SREs in medicated months  F: 31% lower vs M: 35% lower  Odds of SREs 2 years after medication:  F: 14% lower vs M: 19% lower  *Effect sizes could not be calculated* |
| **[78] Biederman et al. (2006)**  Study aim: To compare the efficacy and tolerability of stimulant and non-stimulant treatment for ADHD in school-age girls with ADHD. | ADHD  F 31  M 0  Presentation:  31 ADHD-C | ADHD  F 26  M 0 | 6 – 12 (8.7) | ATX (18 days) vs MAS XR (18 days) | RCT with ITT | MAS XR  Decreased appetite (40.7%)  Upper abdominal pain (29.6%)  Insomnia (25.9%)  Headache (14.8%)  Weight decrease (7.4%)  Anorexia (7.4%)  ATX  Somnolence (28.1%)  Upper abdominal pain (15.6%)  Vomiting (15.6%)  Nausea (12.5%)  Decreased appetite (12.5%).  Headache (9.4%) | **Improvement in behaviour, attention and academic performance**  Deportment  MAS-XR > ATX  Attention  MAS-XR > ATX  Attempting to solve problems  MAS-XR > ATX  Accuracy  MAS-XR = ATX  *Effect sizes could not be calculated* |
| **[80] Wehmeier et al. (2012)**  Study aim: to evaluate treatment differences with respect to health-related quality of life and ADHD symptoms across sexes. | ADHD  F 136  Presentation:  ADHD-C  ADHD-I  ADHD-HI | ADHD  M 658 | F: 6 – 15 (9.6)  M: 6 – 15 (9.7) | ATX (8 – 12 weeks) vs placebo (8 – 12 weeks) vs | Multicenter, open-label, single-arm study | NR | **Various ADHD related behaviours**  Females vs males  Tendency towards behaviours with adverse consequences in general:  F: ATX < P (*d* = 0.57)  M: ATX < P (*d* = 0.49)  Tendency to pertaining to themselves:  F: ATX < P (*d* = 0.64)  M: ATX < P (*d* = 0.45)  Self-content: F < M (*d* = 0.24)  Behaviours disturbing the process of growing up: F < M (*d* = 0.40)  Ability to solve interpersonal conflicts: F < M (*d* = 0.24)  Educational development relative to age: F < M (*d* = 0.51)  Skills in the classroom: F < M (*d* = 0.44)  Connections to classmates: F < M (*d* = 0.39) |
| **[81] Biederman et al. (2002)**  Study aim: To examine effects of non-stimulant drugs on school-aged girls. | ADHD  F 30  M 0  Presentation:  41 ADHD-C  11 ADHD-I  (1 discontinued) | ADHD  F 21  M 0 | 7-13 (9.7) | ATX (9 weeks) versus Placebo (9 weeks) | Placebo-controlled observational study | ATX  Rhinitis 25.8%  Abdominal pain 29.0%  Headache 25.8%  Pharyngitis 19.4%  Decreased appetite 19.4% Vomiting 19.4%  Cough increased 16.1%  Fever 9.7%  Placebo  Rhinitis 38.1%  Abdominal pain 14.3%  Headache 14.3%  Pharyngitis 19.0%  Decreased appetite 19.0%  Nervousness 14.3% Somnolence 14.3%  Nausea 14.3%  Emotional lability 14.3% | **Decrease in symptom severity scores**  ADHD-RS Total score:  ATX > placebo  Hyperactivity:  ATX > placebo  CPRS-R:  ATX > placebo  CGI-S  ATX > placebo  **Decrease in attention impairment scores**  ADHD-RS  Inattentiveness  ATX > placebo  *Effect sizes could not be calculated* |
| **[82] Marchant et al. (2011)**  Study aim: To confirm whether emotional dysregulation and/or gender is associated with treatment response, and to assess when ATX has its full pharmacological effect. | ADHD  F 137  Presentation:  259 ADHD-C  117 ADHD-I  7 ADHD-HI | ADHD  M 247 | NR (42.1) | ATX (8 – 156 weeks) vs placebo (8 – 156 weeks) | Open-label study of two multicenter, double-blind trials. | NR | **Improvement in symptom severity**  Females vs males  Overall:  F > M (*d* = 1.7 vs *d* = 1.0)  Hyperactivity & impulsivity:  F > M, *d* = 0.18  Emotional dysregulation:  F > M, *d* = 0.17  Total score:  F> M (*d* = 1.8 vs *d* = 1.6) |

ADHD-C: Combined-type ADHD, ADHD-HI: Hyperactive/Impulsive-type ADHD, ADHD-I: Inattentive-type ADHD, ADHD-RS: Attention Deﬁcit/Hyperactivity Disorder (ADHD) Rating Scale—IV, (ADHD-RS) Parent Version (DuPaul et al. 1998), ATX: Atomoxetine, *d*: Cohen’s d, dAMP: Dextroamphetamine,ED: emotional dysregulation, F: Females, fMRI: Functional magnetic resonance imaging, ITT: intention to treat analysis, lAMP: Levoamphetamine, LisdexAMP: Lisdexamphetamine, M: males, MethAMP: MethylAmphetamine, MAS-XR: Mixed amphetamine salts extended release, MPH: Methylphenidate, NR: Not reported, P: Placebo, SD: Standard deviation, RCT: Randomised controlled trial, SREs: Substance related events.
