# Supplementary material for: The female side of pharmacotherapy for ADHD—A systematic literature review
Source: PLoS One. 2020 Sep 18;15(9):e0239257. doi: 10.1371/journal.pone.0239257 (PMC7500607; doi:10.1371/journal.pone.0239257)
Supplement: Attachment — Submitted filename: PlosOneSexDifferencesADHDMeds 06-14-20.docx [file pone.0239257.s004.docx]

The authors of this paper describe it as a comprehensive review of prior studies examining differences between the sexes regarding prescription rates of ADHD medications (both stimulant and non-stimulant), or regarding treatment responses and tolerability. The authors seem to have done a reasonable search of the literature, based on their reported methods, but were only able to identify 7 studies regarding differences across sexes in prescription rates, and 14 studies regarding differences across sexes in efficacy/effectiveness out of the 2000+ searched. I found the discussion of treatment effects potentially being moderated by hormonal effects and phases of the menstrual cycle in women especially interesting. The authors make a reasonable argument that investigators should routinely examine and report potential differences by sex in variables regarding treatment response and tolerability. I have several suggestions for how this paper could be improved to make it more suitable for potential publication, including the following:

1. Comparisons of males and females regarding their respective rates and ages of diagnoses, as well as their relative treatment responses and tolerability are both clinically relevant. The differences in rates and ages of diagnoses between females and males seem to reflect societal issues and biases. On the other hand, differences regarding pharmacological responses would reflect biological differences across the sexes regarding pharmacokinetic and pharmacodynamic characteristics. Both are important topics in comparing males and females with ADHD, but in my opinion seem somewhat incongruent topics to combine into a single paper.
2. On page 4, lines 11 to 13, I’m not sure what that sentence is trying to say. Is it that symptoms of ADHD are often associated with emotional dysregulation, which makes their accurate diagnosis harder? I also wonder why they emphasize that inattentive ADHD-IT as associated with emotional dysregulation. My understanding of the literature is that the hyperactive-impulsive symptoms of ADHD are more commonly associated with emotional dysregulation than the inattentive symptoms are.
3. The Introductory section of this paper, from pages 1 to 9, seems quite long, and though it has some good information about ADHD, should be shortened to avoid overshadowing the rest of the paper.
4. Page 15, line 3, the p value is 0.58, which is described as “approaching significance.” Could the authors have meant to report it as p = 0.058?
5. Page 15, line 22, do the authors meet severity “ratings” instead of “rates”?
6. On page 15, at lines 23 to 24, the use of the “after” seems incorrect and confusing. If they say that one relationship was true “after” a certain time frame, the later time frames are also “after” that time frame and don’t have the same relationship between sexes. Changing the word “after” to “at” would eliminate any confusion.
7. On page 16, in line 7, “less” should be “lesser,” and in line 8, the word “adult” should be omitted since the authors have already mentioned the age group as adult, and men and women are adults.
8. On pages 15 to 17, adding p-values the effect sizes (wherever possible) would be helpful.
9. On page 17, line 10, the comma after [77] should be a semicolon or a period.
10. On page 20, lines 6 -10, the authors report that the study results of reference 84 suggested that boys have more robust improvements in both inattentive and hyperactive-impulsive symptoms in at least one study of an extended release MPH. They speculate that such differences are potentially due to girls less substantial improvements on MPH relative to placebo in both inattentive and hyperactive-impulsive symptoms in girls on MPH compared to boys on MPH 84 reference 84could the lesser reduction in hyper-impulsive symptoms in females than males be due to a lesser level of those symptoms in the first place?
11. Also on page 20, the authors comment in line 19 that once daily dosing of MPH may be inappropriate for girls. Was immediate release MPH used in that study? If so, we wouldn’t expect once daily MPH to be optimal?
12. On page 20, lines 20 to 22, the authors summarize findings of two studies of dexAMP, which they incorrectly refer to as a non-stimulant medication (page 20, line 21). They also need to add the reference numbers for those two studies of this medication.
13. On page 22, lines 16 through 19, there seem to be two sentences in a row saying the same thing.
14. There were quite a few typos, misspelled words, and missing or incomplete words. The authors regularly used semi-colons (;) when they should have used commas (,) or vice versa. The sentences were often too long, and structured in ways that made them hard to read and comprehend, without re-reading them. I would encourage the authors to carefully proof-read and perhaps have someone else whose primary language is English also proof read the next draft to reduce such issues, simplify the sentence structure, and improve the papers’ readability and impact. Here are some of the things that needed to be addressed:
    a. Page 2, line 11: should have a comma after girls
    b. Page 3, line 7 “varies” should be “vary”.
    c. Page 3, line 22, sentence would read better if the authors shortened the sentence to say: “Girls tend to be diagnosed…”
    d. Page 3, line 23, put a [ in front of 18,20]
    e. Page 6, line 8, “short-term should be replaced with “short-acting” to go with “long-acting.”
    f. Page 6, line 19, “considerable” should be ”considerably”
    g. Page 6, line 25, “nclude” I believe is meant to be “conclude”
    h. Page 8, line 23: should not capitalize the t in The but do capitalize the N in National.
    i. Sentence starting on bottom of page 9 and ending on page 10’s line 2 is unclear “measure instrument were set.”
    j. Page 12, line 23, I’d suggest switching word “confirmed” with “replicated”.
    k. Page 13, line 18, I’d suggest “nearly diminished” changed to “nearly gone”
    l. Page 15, line 3, p value of “0.58” reportedly approaching significance probably should 0.058
    m. Page 16, line 8, men and women are adults; omit word “adult” in front of them.
    n. Page 17, line 10; immediately after [77] should be a period or “;” not a comma.
    o. Page 17, line 13 and page 18, line 13, Atomoxetine is misspelled.
