# Supplementary material for: The female side of pharmacotherapy for ADHD—A systematic literature review
Source: PLoS One. 2020 Sep 18;15(9):e0239257. doi: 10.1371/journal.pone.0239257 (PMC7500607; doi:10.1371/journal.pone.0239257)
Supplement: Attachment — Submitted filename: Rebuttal letter Reviewer #2.docx [file pone.0239257.s005.docx]

**RE:	PONE-D-19-29154**

**“The female side of pharmacotherapy for ADHD – a systematic literature review”**

**BY Kok et al.**

**Point-to-Point reply to the reviewers’ comments:**

**Reviewer #2**

We thank reviewer #2 for the evaluation of our study and the points of criticism raised. All comments are addressed and, where relevant, referred to the specific page and line(s) in the document ‘Revised Manuscript with Track Changes’, where any changes or additions have also been highlighted.

NB: Due to the considerable shortening of the introduction, page and line numbers now differ. In our replies we mention the new page and line numbers where the adjustments are highlighted.

The authors of this paper describe it as a comprehensive review of prior studies examining differences between the sexes regarding prescription rates of ADHD medications (both stimulant and non-stimulant), or regarding treatment responses and tolerability. The authors seem to have done a reasonable search of the literature, based on their reported methods, but were only able to identify 7 studies regarding differences across sexes in prescription rates, and 14 studies regarding differences across sexes in efficacy/effectiveness out of the 2000+ searched. I found the discussion of treatment effects potentially being moderated by hormonal effects and phases of the menstrual cycle in women especially interesting. The authors make a reasonable argument that investigators should routinely examine and report potential differences by sex in variables regarding treatment response and tolerability. I have several suggestions for how this paper could be improved to make it more suitable for potential publication, including the following:

1. Comparisons of males and females regarding their respective rates and ages of diagnoses, as well as their relative treatment responses and tolerability are both clinically relevant. The differences in rates and ages of diagnoses between females and males seem to reflect societal issues and biases. On the other hand, differences regarding pharmacological responses would reflect biological differences across the sexes regarding pharmacokinetic and pharmacodynamic characteristics. Both are important topics in comparing males and females with ADHD, but in my opinion seem somewhat incongruent topics to combine into a single paper.

We agree that these are two different concepts. Several studies reflect societal issues and biases, while several other studies reflect biological differences. These concepts are however related to each other and in order to provide a complete overview of this topic, we decided to address both in this paper. We have now addressed this issue in the discussion, as part of the limitations section: “ In addition, this review includes both studies that reflect societal issues and biases, and studies that reflect biological differences. Considering these concepts are related to each other, and in order to provide a complete overview of this topic, both concepts have been addressed in the current article.” (p22, line 15-19)

2. On page 4, lines 11 to 13, I’m not sure what that sentence is trying to say. Is it that symptoms of ADHD are often associated with emotional dysregulation, which makes their accurate diagnosis harder? I also wonder why they emphasize that inattentive ADHD-IT as associated with emotional dysregulation. My understanding of the literature is that the hyperactive-impulsive symptoms of ADHD are more commonly associated with emotional dysregulation than the inattentive symptoms are.

Thank you for your valuable comment. We have conducted an extensive literature search on the links between both female biological sex and ADHD-I, and between female sex and emotional dysregulation. A strong link is found in several recent studies between female sex and emotional dysregulation, via the pathway ADHA-I. Hence we describe the sentence in this manner. Some articles that discuss these links include, but are not limited to the reference noted in our manuscript and:

• Hirsch, O., Chavanon, M.L. & Christiansen, H. Emotional dysregulation subgroups in patients with adult Attention-Deficit/Hyperactivity Disorder (ADHD): a cluster analytic approach. Sci Rep 9, 5639 (2019). https://doi.org/10.1038/s41598-019-42018-y

• Retz-Junginger, P., Rösler, M., Müller, R., & Retz, W. (2012). Impact of gender on the utilization of outpatient health service for adult ADHD. Psychiatrische Praxis, 39(7), 345-348.

We have now rephrased the sentence to “Symptoms of ADHD-I, more characteristic of females, are often reflected in mood- or emotional dysregulation making differentiated diagnostics quite difficult.” (p4, line 3-5)

3. The Introductory section of this paper, from pages 1 to 9, seems quite long, and though it has some good information about ADHD, should be shortened to avoid overshadowing the rest of the paper.

We agree, and have reduced the introduction from nearly 7 to 4,5 pages (albeit with pain in our hearts..).

4. Page 15, line 3, the p value is 0.58, which is described as “approaching significance.” Could the authors have meant to report it as p = 0.058?

Correct! Thank you for highlighting this mistake. We have adjusted it accordingly (p13, line 4).

5. Page 15, line 22, do the authors meet severity “ratings” instead of “rates”?

We did indeed, thank you (p13, line 23).

6. On page 15, at lines 23 to 24, the use of the “after” seems incorrect and confusing. If they say that one relationship was true “after” a certain time frame, the later time frames are also “after” that time frame and don’t have the same relationship between sexes. Changing the word “after” to “at” would eliminate any confusion.

Very good point, we have adjusted both occurrences (p13 line 25- p14 line 2).

7. On page 16, in line 7, “less” should be “lesser,” and in line 8, the word “adult” should be omitted since the authors have already mentioned the age group as adult, and men and women are adults.

We have made these adjustments (p14, line 8 & 9).

8. On pages 15 to 17, adding p-values the effect sizes (wherever possible) would be helpful.

We agree. However, on some occasions the effect sizes were reported without p-value. On the occasions where we had sufficient data to calculate the effect size ourselves we reported the p-value, when we did not, no p-value could me noted.

9. On page 17, line 10, the comma after [77] should be a semicolon or a period.

Corrected! (p15, line 10)

10. On page 20, lines 6 -10, the authors report that the study results of reference 84 suggested that boys have more robust improvements in both inattentive and hyperactive-impulsive symptoms in at least one study of an extended release MPH. They speculate that such differences are potentially due to girls less substantial improvements on MPH relative to placebo in both inattentive and hyperactive-impulsive symptoms in girls on MPH compared to boys on MPH 84 reference 84could the lesser reduction in hyper-impulsive symptoms in females than males be due to a lesser level of those symptoms in the first place?

We thank you for your thoughtful point. We have double-checked the data and confirmed that “Both groups (authors: females & males) were equally impaired (authors: at baselines) on the inattention subscale (T score>70), on the subtypes of ADHD (w2(1)>0.831; p¼0.362) and all CBCL subscales.”

Reference: Günther, T., Herpertz-Dahlmann, B., & Konrad, K. (2010). Sex differences in attentional performance and their modulation by methylphenidate in children with attention-deficit/hyperactivity disorder. Journal of child and adolescent psychopharmacology, 20(3), 179-186; p 180, last section)

11. Also on page 20, the authors comment in line 19 that once daily dosing of MPH may be inappropriate for girls. Was immediate release MPH used in that study? If so, we wouldn’t expect once daily MPH to be optimal?

Thank you for your valuable comment. The two studies mentioned in the section you referred to, involved the effects of orally administrated immediate release MPH in both boys and girls with ADHD. The studies found that when given the same dosage of immediate release MPH in the morning, the girls experienced had a stronger effect of MPH earlier in the day but also experienced an earlier decline compared to boys with this disorder. Since boys did not experience an earlier decline, the authors stated it was likely that a once-daily dose of MPH immediate release may be suitable for boys but not so much for girls. We hope we have hereby answered your questions appropriately.

12. On page 20, lines 20 to 22, the authors summarize findings of two studies of dexAMP, which they incorrectly refer to as a non-stimulant medication (page 20, line 21). They also need to add the reference numbers for those two studies of this medication.

Correct! The heading of that particular section of the results was ‘*Stimulant efficacy / effectiveness’* so the study results were fitting but a small mistake was made. We have corrected this mistake, inserted the references and thank you (p18, line 20 & 21).

13. On page 22, lines 16 through 19, there seem to be two sentences in a row saying the same thing.

Thank you, corrected (p20, line 16-18)

14. There were quite a few typos, misspelled words, and missing or incomplete words. The authors regularly used semi-colons (;) when they should have used commas (,) or vice versa. The sentences were often too long, and structured in ways that made them hard to read and comprehend, without re-reading them. I would encourage the authors to carefully proof-read and perhaps have someone else whose primary language is English also proof read the next draft to reduce such issues, simplify the sentence structure, and improve the papers’ readability and impact.

N.B. The primary language of the first author of this manuscript in English. She has noticed before that the use of semi-colons, frequently used in for example Ireland, is less common in other countries. For the purpose of this manuscript, we agree on your comment and have adjusted all points referred to at comment 15 below.

15. Here are some of the things that needed to be addressed:
    a. Page 2, line 11: should have a comma after girls **V**
    b. Page 3, line 7 “varies” should be “vary” **V**
    c. Page 3, line 22, sentence would read better if the authors shortened the sentence to say: “Girls tend to be diagnosed…” Thank you, although we shortened the introduction considerably already, we had not spotted this point (p3, line 14-15).
    d. Page 3, line 23, put a [ in front of 18,20] **V**
    e. Page 6, line 8, “short-term should be replaced with “short-acting” to go with “long-acting.” **V**
    f. Page 6, line 19, “considerable” should be ”considerably” **V**
    g. Page 6, line 25, “nclude” I believe is meant to be “conclude” In shortening our introduction, this sentence has been removed.
    h. Page 8, line 23: should not capitalize the t in The but do capitalize the N in National. **V**
    i. Sentence starting on bottom of page 9 and ending on page 10’s line 2 is unclear “measure instrument were set.”

We have adjusted the relevant sentence to “Due to the scarce body of literature, no strict excluding criteria for the measurement of efficacy / effectiveness were set” (p8, line 3-4). Thank you for your suggestion.

j. Page 12, line 23, I’d suggest switching word “confirmed” with “replicated”. **V**
k. Page 13, line 18, I’d suggest “nearly diminished” changed to “nearly gone” **V**
l. Page 15, line 3, p value of “0.58” reportedly approaching significance probably should 0.058 **V**
m. Page 16, line 8, men and women are adults; omit word “adult” in front of them. **V**
n. Page 17, line 10; immediately after [77] should be a period or “;” not a comma. **V**
o. Page 17, line 13 and page 18, line 13, Atomoxetine is misspelled. **V**

Gratefully yours,

Francien Kok and colleagues
